# Urolithin B: Two-way attack on IAPP proteotoxicity with implications for diabetes

**DOI:** 10.3389/fendo.2022.1008418

**Published:** 2022-12-15

**Authors:** Ana F. Raimundo, Sofia Ferreira, Vânia Pobre, Mafalda Lopes-da-Silva, José A. Brito, Daniel J. V. A. dos Santos, Nuno Saraiva, Cláudia N. dos Santos, Regina Menezes

**Affiliations:** ^1^ iBET, Instituto de Biologia Experimental e Tecnológica, Oeiras, Portugal; ^2^ ITQB-NOVA, Instituto de Tecnologia Química e Biológica António Xavier, Universidade NOVA Lisboa, Oeiras, Portugal; ^3^ iNOVA4Health, NOVA Medical School|Faculdade de Ciências Médicas, NMS|FCM, Universidade Nova de Lisboa, Lisboa, Portugal; ^4^ CBIOS – Universidade Lusófona’s Research Center for Biosciences & Health Technologies, Lisboa, Portugal; ^5^ Universidad de Alcalá, Escuela de Doctorado, Departamento de Ciencias Biomédicas, Madrid, Spain

**Keywords:** amylin, diabetes, IAPP, small molecule, urolithin B

## Abstract

**Introduction:**

Diabetes is one of the major metabolic diseases worldwide. Despite being a complex systemic pathology, the aggregation and deposition of Islet Amyloid Polypeptide (IAPP), or amylin, is a recognized histopathological marker of the disease. Although IAPP proteotoxicity represents an important trigger of β-cell dysfunction and ultimately death, its exploitation as a therapeutic tool remains underdeveloped. The bioactivity of (poly)phenols towards inhibition of pathological protein aggregation is well known, however, most of the identified molecules have limited bioavailability.

**Methods:**

Using a strategy combining in silico, cell-free and cell studies, we scrutinized a unique in-house collection of (poly)phenol metabolites predicted to appear in the human circulation after (poly)phenols ingestion.

**Results:**

We identified urolithin B as a potent inhibitor of IAPP aggregation and a powerful modulator of cell homeostasis pathways. Urolithin B was shown to affect IAPP aggregation pattern, delaying the formation of amyloid fibrils and altering their size and morphology. The molecular mechanisms underlying urolithin B-mediated protection include protein clearance pathways, mitochondrial function, and cell cycle ultimately rescuing IAPP-mediated cell dysfunction and death.

**Discussion:**

In brief, our study uncovered urolithin B as a novel small molecule targeting IAPP pathological aggregation with potential to be exploited as a therapeutic tool for mitigating cellular dysfunction in diabetes. Resulting from the colonic metabolism of dietary ellagic acid in the human body, urolithin B bioactivity has the potential to be explored in nutritional, nutraceutical, and pharmacological perspectives.

## Introduction

Type 2 Diabetes (T2D) is a multifactorial pathology mainly caused by reduction in β-cell function and mass, and insulin resistance (1, 2). Associated with reduced insulin sensitivity is the high insulin production as an overcompensation mechanism (3, 4). Alongside with insulin, there is the co-expression of Islet Amyloid Polypeptide (IAPP), or amylin, a 37-amino acids polypeptide hormone. It is expressed in β-cells as preproIAPP (ppIAPP), an immature form attached to a signal peptide. To reach its mature form, ppIAPP requires cleavage by the same enzymes that process insulin, leading to the overburden of the system and accumulation of immature forms of IAPP. Physiologically, mature IAPP is stored in the secretory granules, being released to the bloodstream in response to glucose. It acts synergistically with insulin to stabilize post-prandial blood glucose, manages gastric emptying, contributes to satiation, and controls adiposity (5–7). However, IAPP is also one of the most amyloidogenic proteins known and its deposits are present in ~90% of individuals with T2D (8, 9). Additionally, immature forms of IAPP that accumulate in the β-cell are also extremely amyloidogenic, contributing to accelerate the aggregation kinetics (10–13). IAPP oligomers cause deleterious effects in virtually every function of β-cells, interfering with numerous molecular pathways (14). Autophagy, a cell mechanism that removes damaged organelles and helps clear toxic proteins, is particularly affected. It is usually upregulated when cells are under stress attempting to remove harmful components and aggregation-prone proteins (15, 16). Another affected cellular mechanism is IAPP-induced increase in oxidative stress with implications in β-cell apoptosis (17). Consequently, alterations in mitochondrial function and metabolism are associated with IAPP.

Molecules potentially interfering with IAPP aggregation process offer a promising therapeutic venue. This pursuit has explored the bioactive properties of (poly)phenols, phytochemicals present in a myriad of foodstuffs, including fruits and vegetables. The potential of (poly)phenols for attenuating IAPP pathological protein aggregation, with implications for degenerative diseases, has been recently reviewed by us (18). In the context of diabetes, (poly)phenols have been described to reduce fasting glucose levels, particularly in combination with antidiabetic medication (19). As for IAPP aggregation, the most described compounds are epigallocatechin gallate (EGCG) (20, 21) and resveratrol (22–24). These were repeatedly reported to inhibit aggregation and even breaking pre-formed fibrils, protecting cell models of IAPP toxicity, abolishing amyloid growth and membrane damage (25). However, these compounds have low bioavailability and are targets of extensive metabolism not only by human cells but also by gut bacteria. This means that the molecule that successfully reaches the target organs is not the one existing in (poly)phenol-rich foodstuffs but their metabolites, thus limiting the translation of the effects found *in vitro* to the human organism. In that view, it is of interest to study these metabolites to overcome the issues of parent compounds (26).

To unveil dietary (poly)phenol metabolites as potential small molecules against IAPP aggregation, we designed a screening strategy for testing a collection of (poly)phenol metabolites. Using *in silico* tools, the molecules were narrowed to urolithins as the class with the best binding scores (*i.e.* most negative Vina score values) to IAPP. Cell-free analysis, using pure IAPP and a versatile eukaryotic model expressing human ppIAPP (27) disclosed urolithin B as the metabolite with best performance. The effects of urolithin B towards the modification of IAPP aggregation process and the modulation of molecular mechanisms underlying cellular responses against IAPP proteotoxicity are herein revealed.

## Materials and methods

### Molecular docking

Molecular docking studies were performed using the software AutoDock Vina (28) and visualized using UCSF Chimera 1.15. The NMR structure of human IAPP was assessed from the Protein Data Bank (PDB) under the reference 2L86. This structure was selected since it represents IAPP in its natively amidated form at physiological pH (29). For simplicity of analysis, only the first conformer in the NMR ensemble was used for docking studies. The ligand structures were obtained from the PubChem database (30). All the structures were minimized and prepared for docking using the feature Dock Prep by adding hydrogens and assigning partial charges. The PDBQT files needed for docking were obtained using the python scripts included in the AutoDock MMGLTools software suit. The search space was defined as 31 x 35 x 20 Å^3^, a minimal rectangular parallelepiped enclosing the full polypeptide chain, to allow a “blind” docking simulation. The ligand-receptor affinity was compared through the AutoDock Vina score, which calculates the affinity of protein-ligand binding (31). Each docking solution was further optimized by manual fitting in COOT (32), followed by structure idealization with REFMAC5 (33), and subsequent analysis in PyMol (Version 2.4.1, Schrödinger, LLC). All structural figures were drawn with PyMol.

A refinement of this initial docking procedure was performed using a smaller docking box with 15x18x15 Å^3^ and centered in the central cavity of the 2L86 structure. Two other similar trials were performed by increasing the exhaustiveness conformational and space searching parameter to 80 or by using the Vinardo scoring function (34).

Another independent docking trial was performed using MOE v2020.0901 (Molecular Operating Environment V2020.0901, Chemical Computing Group Inc, 2022) using default parameters (triangle matcher placement, best 30 ranked with London dG scoring function and then re-scored with the GBVI/WSA dG function) and the docking site defined by the top ranked position of the epigallocatechin 3-gallate molecule (EGCG; the largest one).

### Yeast strains and growth conditions

The strains used in this study are listed in **Supplementary Table 1**. The construct ppIAPP-GFP was previously described (27). For all experiments, cells were grown in synthetic dropout (SD)-glucose medium [0.67% (w/v) yeast nitrogen base (YNB) without amino acids (Difco, United States), 0.77 g/L single amino acid dropout CSM-URA (MP Biomedicals, United States), and 2% (w/v) glucose (Sigma-Aldrich, United States)] for 24 h at 30°C under orbital shaking. Cultures were diluted in fresh medium and, unless stated otherwise, they were incubated under the same conditions until the optical density at 600 nm (OD_600_) reached 0.5 ± 0.05 (log growth phase). The following equation was used to synchronize the cultures: OD_i_ × V_i_ = ODf/[2(t/gt)] × V_f_, where OD_i_ is the initial optical density of the culture, V_i_ is the initial volume of culture, OD_f_ is the final optical density of the culture, t is the time (usually 16 h), gt is the generation time of the strain, and V_f_ is the final volume of culture. Readings were performed in 96-well plates using a Biotek Power Wave XS plate spectrophotometer. Cell cultures were diluted as indicated for each assay. In all experiments, repression or induction of ppIAPP-GFP expression was carried out in SD-glucose medium and SD-galactose [0.67% (w/v) YNB, 0.77 g/L CSM-URA, 2% (w/v) galactose (Sigma^®^, Germany)], respectively.

### Growth curves

For the growth curves, cultures were diluted to OD_600_ 0.05 ± 0.005 in SD-glucose and SD-galactose and incubated at 30°C with orbital shaking for 24 h. Growth was monitored hourly by measuring OD_600_ using a Biotek Power Wave XS Microplate Spectrophotometer (Biotek^®^, Winooski, United States). Model-free spline (nonparametric) and model fitting (parametric) approaches were used to calculate the growth parameters in the R software (35, 36). The package *grofit* (37) was used to adjust a model-free spline and the parameters maximum cell growth (μm) and length of the lag phase (lag time), were estimated from the spline fit. The same package was used to adjust to a model-based curve and the parameters were estimated from the best fit model. The 95% confidence intervals (CIs) were calculated *via* bootstrapping for both model-free spline and model-based fits. The results are represented by the best model curve with 95% CIs for each strain compared to the control strain (not expressing IAPP).

### Flow cytometry assays

Cell cultures were diluted to OD_600_ 0.1 ± 0.01 in SD-galactose and incubated at 30°C with or without the compound in test at the indicated concentrations. To test the involvement of autophagy role, cells were incubated with or without 50 μM urolithin B and 0.5 mM of phenylmethanesulfonyl fluoride (PMSF; Sigma-Aldrich, United States), as indicated. After 12 h of induction, cells were incubated with propidium iodide (PI; Merck, Darmstadt, DE) at a final concentration of 5 μg/mL for 30 min at 30°C under orbital agitation and protected from light. Results were expressed as a percentage of PI-positive cells. To test the proteosome role, cells were incubated with or without 50 μM urolithin B, 0.003% (v/v) SDS and 15 μM MG132, as indicated. For the reactive oxygen species monitoring, cells were incubated with 4 µg/mL of dihydroethidium (DHE; Carlsbad, CA, USA) in the dark for 30 min, with orbital shaking and cells were washed twice with PBS. Results were expressed as the mean fluorescence intensity (MFI) of the cells. For the mitochondria membrane potential test, after 12 h of incubation with or without 50 μM urolithin B, cells were incubated with 500 nM MitoTracker Deep Red FM (Thermo Scientific, Waltham, MA USA) for 30 min at 30°C with orbital shake in the dark and cells were then washed twice with PBS. Results were expressed as the MFI of the cells. Flow cytometry assays were performed using a CyFlow Cube 6 (Sysmex Partec GmbH, Goerlitz, Germany). Data analysis was performed using FlowJo software (Tree Star Inc., San Carlos, CA, USA). A minimum of 100,000 events were collected for each experiment. For monitoring cell cycle progression by DNA content, cells were fixed in 70% ethanol on ice for 2 h and were posteriorly treated with 200 mM Tris-HCl (Tris-buffered saline solution - Hydrochloric acid) pH 7.5 containing 0.1 mg/mL RNase A solution, at 37°C overnight. For DNA staining, cells were incubated with PI containing solution [200 mM Tris-HCl (Carl Roth GmbH, Germany) at pH 7.5, 211 mM sodium chloride (NaCl, PanReac Applichem, Germany), 78 mM magnesium chloride (MgCl_2,_ Merck, Darmstadt, DE)], 50 μg/mL PI (Merck, Darmstadt, DE)] for 30 min under the same conditions as mentioned above. After cell sonication (20% intensity, 10 s), flow cytometry analysis was performed using a BD FACS Calibur Flow Cytometer (BD Biosciences, San Jose, CA, USA). Data acquisition and analysis were performed using CellQuest^®^ (BD Biosciences, San Jose, CA, USA) and FlowJo^®^ software, respectively. A minimum of 50,000 events were collected for each experiment.

### *In vitro* aggregation assays

Pure IAPP protein was acquired from Pepmic (Suzhou, China). Protein preparations were done as previously described (38) with minor modifications. In short, 1 mg of protein was resuspended in 1,1,1,3,3,3-hexafluoro-2-propanol (HFIP) at 100 μM, allowed to solubilize at room temperature for at least 5 h, and aliquoted. Proteins samples were then snap frozen in liquid nitrogen and put overnight in a lyophilizer until all the solvent had been removed. Lyophilized protein samples were stored at -80°C until use. For the aggregation studies, proteins were resuspended at a final concentration of 20 μM in 20 mM Tris-HCl buffer at pH 7.4, with or without 50 μM urolithin B, and incubated at 25°C. Aliquots were taken from the solution at indicated time points and stored at -80°C until analysis.

### Thioflavin T assays

Aliquots of 100 μL from the aggregation assays were collected at the indicated time points and added to white 96-well plates (Thermo Scientific, Waltham, MA USA) with 74 μM thioflavin-T solution, allowing incubation for 5 min in the dark. Fluorescence was measured using a SpectraMax i3X (Molecular Devices, San Jose, CA, USA) at 445 nm excitation and 485 nm emission.

### Transmission electron microscopy

For transmission electron microscopy (TEM) assays, 5 μL of each sample was incubated on glow-discharged carbon coated copper grids for 2 min before washing 10 times with dH_2_O. 2% uranyl acetate was used to negatively stain the samples for 2 min before imaging on the Tecnai G2 80-200kv.

### Confocal microscopy

Cell cultures were diluted to OD_600_ 0.1 ± 0.01 in SD-galactose and incubated at 30°C with or without 50 μM urolithin B for 12 h. Cells were collected by centrifugation at 3000 g for 3 min and resuspended in PBS. For the mitochondrial membrane potential experiments, cells were incubated with 500 nM MitoTracker Deep Red FM (Thermo Scientific, Waltham, MA USA) for 30 min at 30°C with orbital shaking, in the dark. Cells were washed twice with PBS and the microscopy slides were prepared using 4 μL of cell suspension mixed with 4 μL of low melting point agarose. GFP and MitoTracker fluorescence was acquired using a Zeiss LSM980 confocal microscope. The number of GFP positive (GFP+) cells, cells with aggregates and determination of aggregate area were monitored in at least 800 cells for each condition using Fiji-ImageJ1.51j8 (39).

### Immunoblotting assays

Cell cultures were diluted to OD_600_ = 0.1 ± 0.01 in SD-galactose and incubated at 30°C for 12 h with or without 50 μM urolithin B. Cells were collected by centrifugation for 4 min at 2,500 g, the pellets were resuspended in TBS [Tris 2.4 g/L (Carl Roth GmbH, Germany), 8 g/L NaCl (PanReac Applichem, Germany), pH 7.6] supplemented with protease and phosphatase inhibitors, cells were disrupted with glass beads (3 cycles of 30 s in the vortex and 5 min on ice), and cells debris were removed by centrifugation at 700 g for 3 min. Total protein was quantified using the MicroBCA kit (Thermo Fisher Scientific, United States) according the manufactures’ instructions. Samples were incubated at 95°C for 10 min before sodium dodecyl sulphate-polyacrylamide gel electrophoresis (SDS-PAGE). Ten micrograms of total proteins were loaded and resolved in Mini-Protean TGX Gels (Bio-Rad, United States). Gels were transferred to Polyvinylidene fluoride (PVDF) membranes using the Trans-Blot^®^ SD semi-dry transfer system (Bio-Rad, United States). Membranes were activated with methanol and blocked for 1 h at room temperature with 5% (w/v) bovine serum albumin (BSA, Sigma-Aldrich, United States) dissolved in TBS-T [Tris 2.4 g/L (Carl Roth GmbH, Germany), 8 g/L NaCl (PanReac Applichem, Germany), and 0.1% (v/v) Tween 20 (Sigma-Aldrich, United States)]. The primary antibodies against GFP (Neuromabs, California, United States), IAPP (Sigma-Aldrich, United States), and Pgk1 (Invitrogen, United States) were probed overnight at 4°C. The membranes were then washed 6 × 5 min in TBS-T and incubated with the appropriated secondary antibody for 1 h at room temperature. The membranes were washed 3 × 10 min in TBS-T and incubated with ECL™ Prime Western Blotting System (GE Healthcare, United States). Images were acquired using Odyssey^®^ Fc Imaging System (LI-COR Biosciences) and analyzed using Image Studio^®^ software.

### Filter trap assays

Protein samples from the aggregation assays were diluted in Tris-buffered saline (TBS) 1% (v/v) SDS and loaded onto a pre-equilibrated nitrocellulose membrane (0.22 μm) (GE Healthcare, Chicago, IL, USA) in a slot blot apparatus. Samples were allowed to pass through the membrane by vacuum, and the slots were washed twice with TBS 1% (v/v) SDS. Immunoblotting was performed following standard procedures using anti-IAPP antibody. Images were acquired using an Odyssey^®^ Fc Imaging System (LI-COR Biosciences) and Image Studio^®^ software.

### Triton solubility analysis

Triton soluble and insoluble fractions were separated as described (40). Briefly, 200 μg of total protein was incubated with 1% Triton X-100 for 30 min on ice. The protein was centrifuged at 15,000 g for 60 min at 4°C. The supernatant was considered the soluble protein fraction and collected. The pellet was resuspended in 40 μL of 2% sodium dodecyl sulphate (SDS) Tris-HCl buffer pH 7.4 by pipetting and 10 s of sonication and considered the insoluble fraction. Equal volumes of soluble and insoluble fractions were loaded and resolved by SDS-PAGE as described above.

### Size exclusion chromatography

Total protein lysates from cells incubated for 12 h with or without 50 μM urolithin B were obtained as indicated and centrifuged at 10,000 g for 10 min to remove insoluble particles. Three μg of total protein was diluted in 2 mL of TBS buffer and loaded on a Superose 6 10/300 GL column (GE Healthcare, Uppsala, Sweden) using an AKTA system (GE Healthcare, Uppsala, Sweden). Samples were eluted with PBS [1 mM potassium phosphate, 155 mM NaCl, 2.9 mM sodium phosphate], pH 7.4 at a flow rate of 0.5 mL/min, and the UV absorbance was monitored at 280 nm. To estimate the molecular weight of the protein samples, Gel Filtration HMW Calibration Kit (GE Healthcare, Uppsala, Sweden) plus Ribonuclease (PanReac AppliChem, Spain) was used. Fractions of 500 μL were collected, precipitated overnight at 4°C in trichloroacetic acid (TCA; Sigma-Aldrich, United States), washed three times in acetone, resuspended in a protein sample buffer [0.12 M Tris-HCl, pH 6.8, 9% (v/v) β-Mercaptoethanol, 20% (v/v) glycerol, 4% (w/v) SDS, and 0.05% (w/v) Bromophenol Blue], and resolved by SDS-PAGE using Mini-Protean TGX Gels (Bio-Rad, United States). Immunoblottings were performed as indicated.

### Protein clearance assays

For clearance experiments, after 12 h of induction with or without 50 μM urolithin B, the cells were centrifuged, washed in PBS, resuspended in 2% (w/v) glucose SD liquid media (to repress IAPP expression) and incubated at 30°C, with shaking, for additional 12 h. The levels of IAPP were determined by immunoblotting at 12 h of induction (corresponding to 0 h of clearance) and at 12 h of clearance.

### Transcriptomic analysis

Cell cultures were diluted to OD_600_ = 0.1 ± 0.01 in SD-galactose and incubated at 30°C for 12 h with or without 50 μM of urolithin B. RNA was extracted using Roche High Pure RNA Isolation Kit, following the manufacturer’s instructions, and quantified using the Nanodrop ND-2000C (Thermo Scientific, Waltham, MA, USA). Biological triplicates were used for each condition. Library preparation and sequencing was done at the Genomics Facility of Instituto Gulbenkian da Ciência, Portugal, using the SMART-SEQ2 protocol, adapted from (41) and an Illumina NextSeq500 platform (single end, 75-bp read length, 20 M reads), respectively. RNA-Seq data was analyzed following the workflow described in (42). In summary, the RNA-Seq data quality was confirmed using fastQC program. The reads were mapped against *S. cerevisiae* genome (GCA_000146045.2 downloaded from NCBI genome database) using Bowtie2 program (43). The mapping files were sorted by genomic position using the Samtools (44) and the quantification of the transcripts’ expression was done using the featureCounts program (45). The differential expression analysis was done with the R package edgeR (46). All transcripts with a False Discovery Rate (FDR) correction of the p-value lower than 0.05 were considered as significant and results were further filtered using the expression values (LogCPM) higher than 3 and a fold-change between two samples higher than two. The functional annotation was performed using GeneCodis3 (47).

### Quantitative real-time PCR

RNA was extracted as indicated above. DNA synthesis was performed using Roche Transcriptor First Strand cDNA Synthesis kit according to the manufacturer’s instructions. cDNA was diluted 1:100, and quantification of target genes was performed in a Light Cycler 480 Multiwell Plate 96 (Roche) using the Light-Cycler 480 SYBR Green I Master Kit (Roche) and the oligonucleotide primers listed in **Supplementary Table 2** at a final concentration of 5 μM. Reactions were performed in duplicate in a final volume of 10 μL. Cycle’s threshold (Ct’s) and melting curves were determined using Light Cycler 480 software, version 1.5 (Roche), and results were processed using relative quantification method for relative gene expression analysis. Gene expression data were normalized using *actin* and *PDA1* as internal controls.

### Statistical analysis

Statistical analysis was carried out using GraphPad Prism 9 software. Data are mean ± SD of at least three independent biological replicates.

## Results

### Screening of (poly)phenol metabolites potentially interacting with IAPP

**Figure 1:**
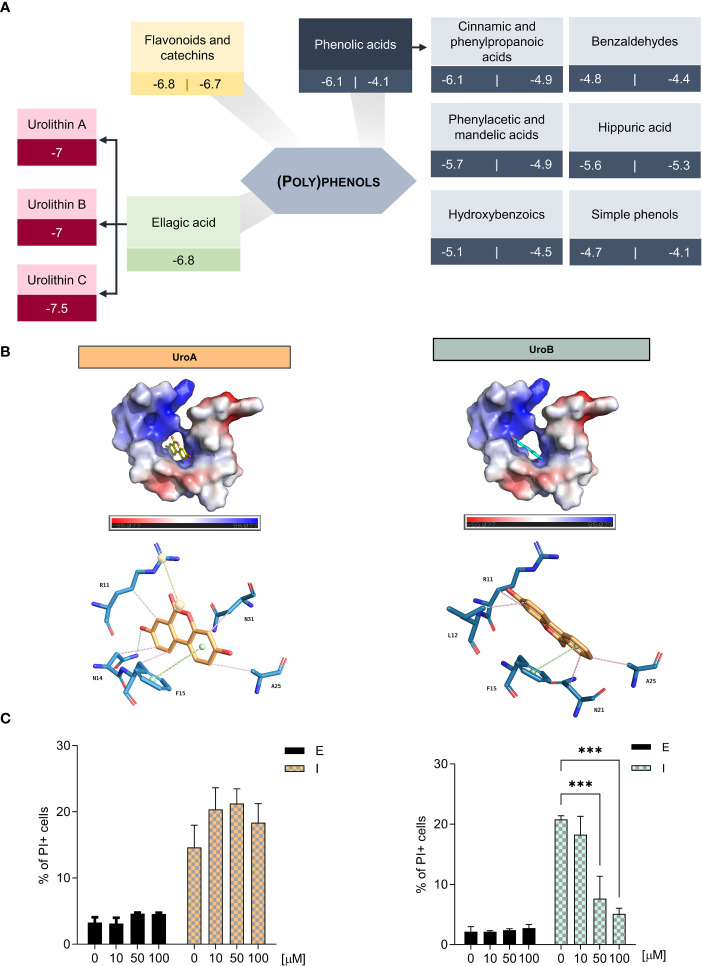
Urolithin B is the (poly)phenol metabolite with better potential for hampering IAPP aggregation **(A)** Docking simulations of IAPP and (poly)phenols by AutoDock Vina reveals urolithins as the best performing molecules. **(B)** Vacuum electrostatics of urolithin A (UroA) and urolithin B (UroB) docking in IAPP using Pymol (upper panel). Predicted bond analysis of UroA and UroB with IAPP amino acid residues (lower panel). Green – π-stacking. Pink – Hydrophobic interactions. Blue – hydrogen bonds. Yellow – Salt bridges. **(C)** The frequency of propidium iodide (PI) positive cells was assessed by flow cytometry in cells expressing ppIAPP and the respective control in the presence of UroA (left) and UroB (right). The values represent mean ± SD from at least three independent experiments. Statistical differences are denoted as ^***^ *p* < 0.001 vs. the control condition. E – cells containing the empty vector. I – cells expressing ppIAPP.

We hypothesized that the binding of (poly)phenols to IAPP would stabilize the monomers, decrease aggregation and amyloidogenicity, thereby avoiding the deleterious consequences of IAPP proteotoxicity. Thus, we defined a strategy to screen a collection of (poly)phenol metabolites. Using the NMR structure of reference 2L86 (PDB), which represents IAPP in its natively amidated form at physiological pH (29), we performed docking studies to identify potential interactions between IAPP and the (poly)phenol metabolites. Starting with approximately 60 molecules (**Supplementary Table 3**), we used the Vina score calculated by the AutoDock software as a measure of the affinity between protein and ligand. To set the limits for Vina score values, we performed simulations with molecules described to interfere with IAPP aggregation *in vitro*: epigallocatechin 3-gallate (EGCG), resveratrol, and myricetin (21, 22, 48), and with molecules without known interaction with IAPP, e.g., aspirin and inositol (49, 50) (**Supplementary Table 4**). Urolithin A, B and C were scored the highest, with as good or better Vina scores as the positive controls (**Figure 1A**).

We characterized further urolithin A, B and C interactions with IAPP by PyMol structure idealization with REFMAC5. The three metabolites fit into the hydrophobic pocket created by the two alpha-helices of the polypeptide chain. Despite having very similar structures (**Supplementary Figure 1A**), which differ only by the presence of additional hydroxyl groups, the molecules interact differently with IAPP (**Figure 1B** and **Supplementary Figure 1B**). Of note, unlike the others, urolithin C does not interact with Phe15 by π-π stacking (**Supplementary Figure 1B**). This type of bond is reported to be crucial to stabilize the aromatic side chains, particularly of Phe15, which is essential for IAPP self-association and aggregation (51, 52). Therefore, only urolithin A and B were further pursued.

To refine the initial blind docking calculation, three other docking analysis were performed using the preferred binding site exposed by the blind docking and, also, in agreement with the prediction by MOE’s “Site Finder”. First, a new docking using this site was performed along with another using the exhaustiveness parameter one order of magnitude larger. All these Vina predictions were largely indistinguishable providing the same conclusions (compounds tested: aspirin, inositol, EGCG, myricetin, resveratrol and urolithin A, B). Another Vina docking using the Vinardo scoring function was performed, also providing a clear separation between the predictions for active and inactive molecules with similar predictions for the most active molecules (**Supplementary Table 4**).

Another independent docking was performed with MOE that uses a different algorithm and scoring functions. The results and specially the docking poses for urolithin A and B are consistent with the ones predicted by Vina with the inactive molecules, specially the smallest polyhydroxy tested (inositol) with the worst predicted binding performance.

The PDB structure 6Y1A when compared with the 2L86, used in the docking, clearly shows that in the amyloid fibril structure formation process both helices are destroyed allowing the fibril to grow through beta sheet interactions. This process is enabled by the membrane hydrophobic environment through changes in the delicate hydrophilic-hydrophobic balance responsible for the 2L86 conformation with the hydrophobic Phe15 playing an important role (51, 52). From the analysis of all the data obtained, a trend emerges pointing to a possible mode of action of these molecules.

Vina predicts a similar binding for urolithin A and C but different than urolithin B. Interestingly, the latter has a binding similar to myricetin. Moreover, the binding for EGCG predicted by MOE has similar contacts since all these three molecules interact with Phe15 by π- π stacking or arene-H interactions. Therefore, it seems plausible that the stabilization of Phe15 along with the stabilization of the internal hydrophobic pocket can be one mode of action to stabilize the structure by preventing the break of alpha-helical turns. In this regard, both the EGCG and myricetin make a bridge between Phe15 and Arg11 through hydrogen bonds with the guanidine group. Finally, when the surface of these molecules is represented by the local hydrophilic/lipophilic characteristics, it is clear that all molecules match the hydrophilic and hydrophobic areas of the docking pocket and, although the EGCG embraces both sides of moon-shaped pocket, it is urolithin B that although being a much smaller molecule closely matches its surface lipophilic characteristics with the pocket.

Nevertheless, urolithin A and B are the end aglycone metabolites of ellagic acid metabolism and have previously reported positive effects towards diabetes (53–56). For these reasons, urolithin A and B were chosen to move forward.

SwissADME (57) was used to evaluate the likelihood of urolithin A and B to be used also as therapeutic small molecules in nutraceuticals and pharmacological perspectives (**Supplementary Table 5**). The predictions indicate that both are moderately soluble in water, with high absorption in the gastrointestinal tract and no violations of “druglikeness” rules, indicating a good potential for the application in therapeutics.

### Urolithin B performs better in the cellular *milieu*

The cellular effects of the urolithin A and B towards IAPP proteotoxicity were next tested in the yeast model of IAPP aggregation (27). Here, ppIAPP is expressed in a inducible fashion and suffers partial processing by endogenous convertases, allowing the accumulation of both mature and immature IAPP forms, as seen in pathological conditions in humans. In this model, a range of urolithin A and B concentrations between 10 and 100 µM was added to the cell culture concomitantly with the induction of ppIAPP-GFP expression. Cell viability was monitored by flow cytometry using PI staining. Urolithin A had no effect in cell viability in any of the tested concentrations (**Figure 1C**, left panel). To overcome possible permeability limitations conferred by the yeast cell wall, urolithin A was tested in the presence of SDS (**Supplementary Figure 1C**). Despite that, no differences in cell viability were seen indicating that urolithin A does not exert protective activity against ppIAPP proteotoxicity in the cellular *milieu* at least in the conditions tested. In contrast, treatment with urolithin B reduced significantly the toxicity caused by ppIAPP expression at 50 and 100 µM (**Figure 1C**, right panel) as the positive control EGCG at 50 µM (**Supplementary Figure 1D**). Testing urolithin B bioactivity against other aggregation-prone proteins such as huntingtin in yeast models failed to confer protection (data not shown).

### Urolithin B interferes with protein aggregation in cell-free and yeast systems

**Figure 2:**
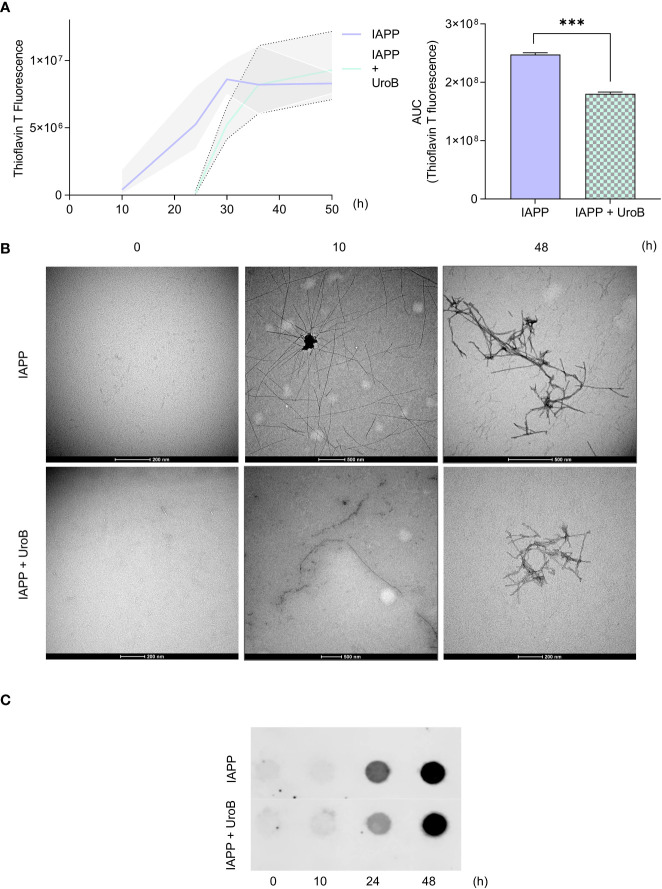
Urolithin B alters the size and structure of IAPP aggregates in cell free system. **(A)** Thioflavin T (ThT) staining of 20 µM pure IAPP protein aggregation with or without 50 µM urolithin B (UroB) shows different aggregation patterns (left panel). Area under the curve of ThT curves (right panel). The values represent mean ± SD from at least three independent experiments. Statistical differences are denoted as ^***^ *p* < 0.001 vs. the control condition. **(B)** Transmission Electron Microscopy of pure IAPP aggregated in the presence or absence of UroB. **(C)** Filter trap assay of samples taken from IAPP aggregation at the indicated time points. Representative images are shown.

To get insight into the mode of action of urolithin B, its ability to directly interfere with IAPP aggregation in cell-free systems was assessed. For that, pure IAPP containing the post-translational modifications found *in vivo* (COOH terminal amidation and disulfide bridge between residues 2 and 7) was allowed to aggregate in the presence or absence of 50 µM urolithin B and monitored through thioflavin T staining (**Figure 2A**). IAPP alone recapitulated the expected pattern of aggregation consisting of a lag phase followed by a rapid exponential growth and a plateau (38). Remarkably, in the presence of urolithin B, the lag phase is visibly prolonged (from 8 to 24 h). Using thioflavin T signal as a measurement of the amount of amyloid fibrils, the area under the curve (AUC) of thioflavin T staining provides information regarding fibrils formed in each condition. As predicted, AUC is reduced when the protein is incubated with urolithin B.

To further characterize the size and morphology of IAPP oligomeric species and fibrils, protein samples were subjected to TEM and filter trap assays. TEM images show that after 10 h of incubation, untreated IAPP presented as complex structures with long and ramified arms. In the presence of urolithin B, IAPP species resemble fibrils composed of globular units with less complex arrangements. As indicated by the ThT assay, complex ramified assemblies with distinct structure were seen in both conditions at 48 h (**Figure 2B**). Consistently, IAPP aggregated in the presence of urolithin B showed a weaker signal than IAPP alone in the filter trap assays until 24 h, indicating that the species formed in this condition are smaller (**Figure 2C**). The results unequivocally show that urolithin B interacts with IAPP, interfering with the aggregation pattern and the overall structure of the amyloid fibrils.

**Figure 3:**
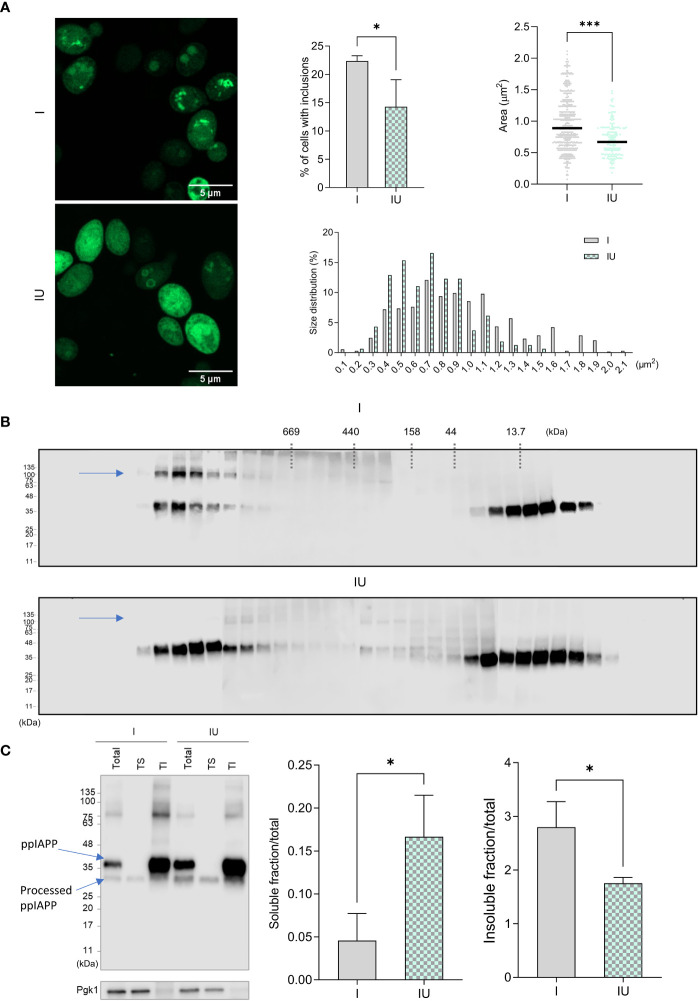
Urolithin B changes the size and the chemical properties of IAPP aggregates in a cellular milieu.**(A)** Confocal microscopy of yeast cells expressing ppIAPP in the presence or absence of 50 µM urolithin B (UroB) indicating the number of cells with inclusions (upper left panel), the area of the inclusions (upper right panel), and their distribution in size (lower panel). **(B)** Size Exclusion Chromatography of protein extracts from cells expressing ppIAPP with or without 50 µM urolithin **(C)** Separation of protein extract according to their solubility in Triton X-100. The values represent mean ± SD from at least three independent experiments. Statistical differences are denoted as ^*^ *p* < 0.05 vs. the control condition. I, cells expressing ppIAPP; IU, cells expressing ppIAPP incubated with UroB. TS, Triton soluble fraction. TI, Triton insoluble fraction. Pgk1, Phosphoglycerate kinase 1. Representative images are shown. Statistical differences are denoted as *p < 0.05, ***p < 0.001 vs. the control condition.

The impact of urolithin B on IAPP aggregation was further evaluated in a yeast model (27). This model is characterized by the accumulation of well-defined intracellular aggregates of ppIAPP-GFP as shown by confocal fluorescence microscopy (**Figure 3A**). In the presence of urolithin B, IAPP aggregates are much less defined, with an even distribution of GFP signal throughout the cell. Importantly, amongst the cells receiving urolithin B, there were less cells with clear, defined inclusions (**Figure 3A**, middle panel). As for the size of the aggregates, the area of the inclusions was considerably smaller, and the size distribution was shifted towards smaller sizes (**Figure 3A**, right panels). We then fractionated the protein extracts by SEC and found a stronger signal in low molecular weight species, presumably low-order oligomers and monomers, suggesting these were more abundant in urolithin B-treated cells (**Figure 3B**). Noteworthy, the SDS-resistant high molecular weight species (blue arrow) disappeared in these cells. This may indicate that not only the size, but also the biochemical properties, of IAPP species are altered by urolithin B. To evaluate this, total protein extracts were separated according to their solubility in Triton X-100, which revealed that the amount of IAPP found in the soluble fraction is higher in urolithin B-treated cells than in control cells whereas the amount of IAPP found in the insoluble fraction is higher in untreated cells (**Figure 3C**).

### Transcriptomics shows the involvement of clearance and metabolism pathways in urolithin B-mediated protection

**Figure 4:**
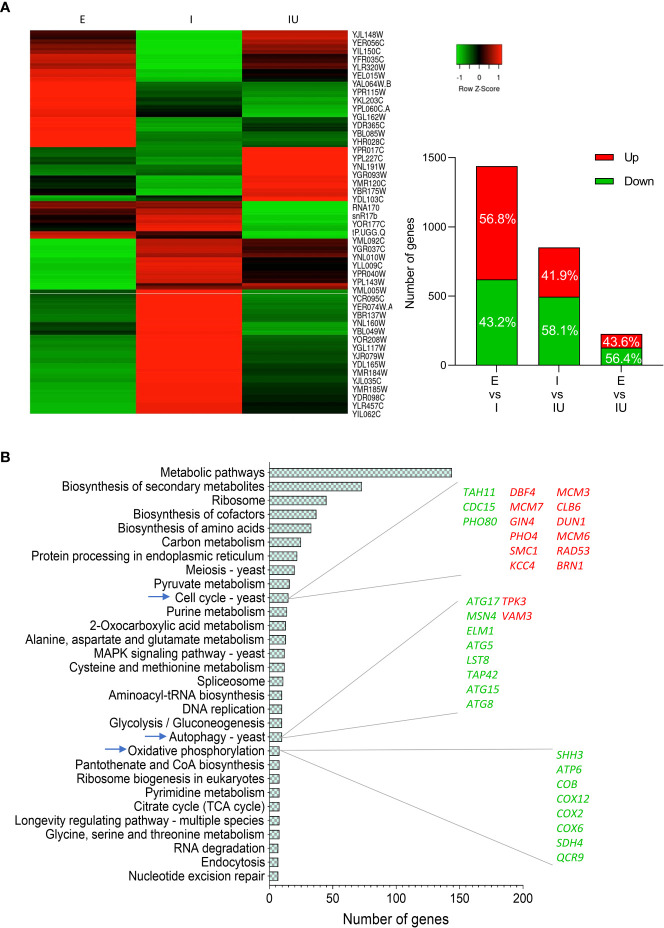
Urolithin B induces a massive transcriptomic change in IAPP-expressing cells **(A)** Heatmap of differently expressed genes of cells expressing ppIAPP with or without 50 µM urolithin B (UroB) and the control expressing the empty vector (left panel). Number of differently expressed genes and distribution in up and downregulation between each comparison (right panel). **(B)** Functional enrichment analysis obtained from GeneCodis of differently expressed genes between cells expressing ppIAPP with or without 50 µM UroB. The list of genes up and down regulated of the explored pathways is also depicted. E, cells containing the empty vector; I, cells expressing ppIAPP; IU, cells expressing ppIAPP incubated with UroB.

To investigate the potential role of urolithin B on cellular homeostasis upon IAPP proteotoxic insult, we performed high-throughput transcriptomics analysis by RNAseq. Comparison of the transcriptomic profile of control cells incubated or non-incubated with urolithin B revealed more than 300 genes significantly altered (**Supplementary Figure 2A**) indicating that urolithin B modulates a genetic reprograming regardless of IAPP burden. Thus, urolithin B emerges as a potential bifunctional molecule preventing the formation of toxic IAPP aggregates and modulating cellular pathways. As for the transcriptional response underlying ppIAPP expression, more than 1,400 genes were significantly altered when compared to the control cells, most of them being upregulated. On the other hand, comparison of the transcriptional profile of ppIAPP-expressing cells with or without urolithin B shows about 850 genes significantly altered, mostly downregulated (**Figure 4A** and **Supplementary Figure 2**). We next compared the transcriptome of control cells with that of cells expressing ppIAPP and incubated with urolithin B. Only 227 genes were significantly different between the two conditions, suggesting that urolithin B attenuates the changes caused by ppIAPP.

The differences found in the transcriptomics were validated by quantitative real-time PCR (qRT-PCR). Transcripts with the highest fold change from the enriched pathways (that were differentially expressed in at least one of the comparisons) were selected, and the fold change was determined by RT-qPCR and compared with the values obtained with the RNAseq (**Supplementary Table 5**).

We then performed pathway enrichment analysis of the genes significantly altered between ppIAPP-expressing cells challenged or not with urolithin B using the GeneCodis database (**Figure 4B**). Not surprisingly, the data showed that several pathways were significantly enriched, most of them previously associated with IAPP-induced toxicity, and a great part related to metabolic alterations.

### Autophagy is essential for urolithin B to overcome IAPP proteotoxicity

**Figure 5:**
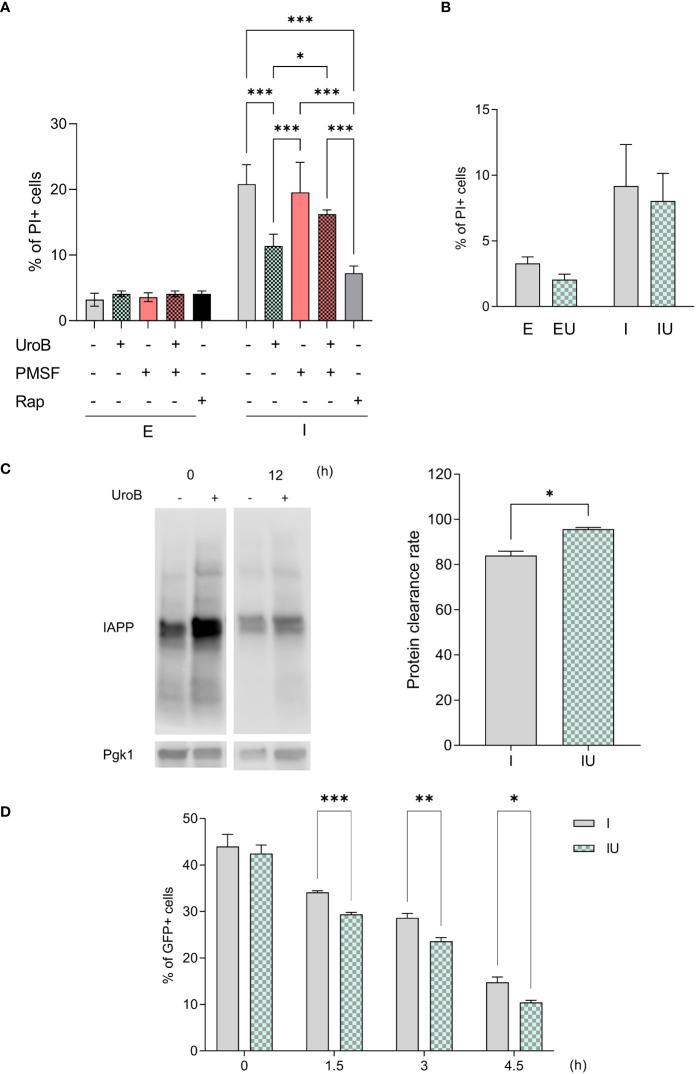
Autophagy and protein clearance contribute to urolithin B-mediated protection. **(A)** The frequency of propidium iodide (PI) positive cells assessed by flow cytometry in cells expressing ppIAPP and the respective control in the presence of 50 µM urolithin B (UroB), the autophagy blocker phenylmethylsulfonyl fluoride (PMSF), or the autophagy inducer rapamycin (Rap); and **(B)** in cells lacking the *ATG8* gene. **(C)** Protein clearance as assessed by immunoblotting. **(D)** The frequency of GFP positive cells by means of flow cytometry at the indicated time points after protein expression blockage. The values represent mean ± SD from at least three independent experiments. Statistical differences are denoted as ^**^ *p* < 0.01, and ^***^ *p* < 0.001 vs. the control condition. E, cells containing the empty vector; I, cells expressing ppIAPP; EU, cells containing the empty vector incubated with UroB; IU, cells expressing ppIAPP incubated with UroB; Pgk1, Phosphoglycerate kinase 1. Statistical differences are denoted as *p < 0.05, **p < 0.01, and ***p < 0.001 vs. the control condition.

Autophagy was unveiled as one of the enriched pathways in the transcriptome of ppIAPP-expressing cells treated with urolithin B in accordance with the relevance of this pathway for IAPP toxicity and β-cell dysfunction in diabetes (16, 58). We therefore used chemical and genetic approaches to block autophagy and to investigate its impact on urolithin B-mediated protection. The proteinase B inhibitor phenylmethylsulfonyl fluoride (PMSF), which causes the accumulation of autophagic bodies in the vacuole (59), was used to inhibit autophagy and IAPP cytotoxicity was monitored by PI staining and flow cytometry. As depicted in **Figure 5A**, urolithin B-mediated protection against IAPP proteotoxicity is lost in the presence of PMSF. Remarkably, urolithin B provides the same level of protection in terms of cell toxicity as the autophagy inducer rapamycin in reducing the deleterious effects of IAPP. We then used a genetic model expressing ppIAPP but bearing the deletion of *ATG8.* The gene encodes a ubiquitin-like protein conjugated to phosphatidylethanolamine (PE) with a role in membrane fusion during autophagosome formation, an ortholog of the mammalian microtubule-associated protein 1A/1B-light chain 3 (LC3). As observed when blocking autophagy chemically, urolithin B conferred no protection in this genetic background (**Figure 5B**). Together with the transcriptomics data, these results point out the role of autophagy in urolithin B-mediated protection against IAPP proteotoxicity.

Autophagy is well known to contribute to the clearance of toxic protein aggregates (60, 61). Taking advantage of the on-off ppIAPP expression system in yeast (27), ppIAPP expression was induced for 12 h in the presence or absence of urolithin B, after which it was rapidly shut down. Clearance was then assessed in both conditions by comparing IAPP protein levels at time point 0 and 12 h after IAPP expression blockage. The data revealed that cells exposed to urolithin B during ppIAPP expression window clear it at a faster rate than untreated cells (**Figure 5C**). Since ppIAPP constructs were fused to GFP (27), IAPP clearance was also followed throughout time after ppIAPP-GFP expression blockage by monitoring the number of GFP+ cells by flow cytometry. The quantity of GFP+ cells were similar immediately after blocking GFP-IAPP but decreases faster in cells incubated with urolithin B (**Figure 5D**). Altogether, the data suggest an increased clearance of IAPP in the presence of urolithin B.

### Urolithin B conveys protection to IAPP-induced mitochondrial damage and dysfunction

**Figure 6:**
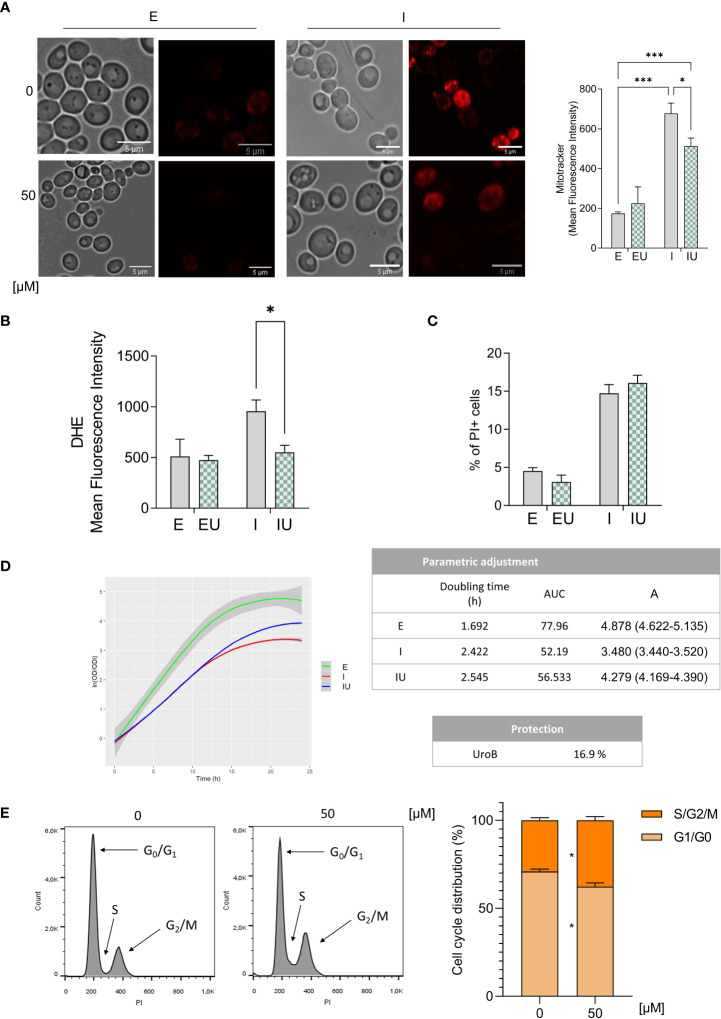
Mitochondrial function, oxidative stress and growth rate are affected by urolithin B treatment.**(A)** Confocal microscopy images of cells expressing ppIAPP and the respective control in the presence of 50 µM urolithin B stained with Mitotracker Dye (left panel). Mean Fluorescence Intensity of Mitotracker dye was assessed by flow cytometry in cells expressing ppIAPP and the respective control (right panel). **(B)** Mean Fluorescence Intensity of dihydroethidium (DHE) was assessed by flow cytometry in cells expressing ppIAPP and the respective control in the presence of urolithin **(C)** The frequency of PI positive cells was assessed by flow cytometry in cells lacking the *yap1* gene and expressing ppIAPP, and the respective control in the presence of 50 µM urolithin **(D)** Growth curve of cells expressing ppIAPP in the presence of 50 µM urolithin B and the respective control measured by OD_600_ during 24 h (left panel). Parametric adjustments of the growth curves and calculated protection (right panel). **(E)** Cell cycle analysis through PI staining through flow cytometry in cells expressing ppIAPP in the presence of 50 µM urolithin B (left panel). Quantitative measurement of relative cell cycle phase (right panel). The values represent mean ± SD from at least three independent experiments. Statistical differences are denoted as ^***^ *p* < 0.001 vs. the control condition. UroB – Urolithin (B) PI, propidium iodide; E, cells with the empty vector; I, cells expressing ppIAPP; IU, cells expressing ppIAPP incubated with UroB. Statistical differences are denoted as *p < 0.05, and ***p < 0.001 vs. the control condition.

Previous reports have connected mitochondrial dysfunction with metabolic dysregulation and insulin secretory failure (62). Oxidative phosphorylation, pointed by the transcriptomics in urolithin B-mediated protection, is directly dependent of mitochondrial membrane potential. Thus, Mitotracker, a dye which accumulates in the mitochondria in a membrane potential-dependent manner was used to assess the impact of ppIAPP proteotoxicity on mitochondria and to evaluate in a semi-quantitative manner the protective potential of urolithin B towards the maintenance of mitochondrial mass and activity (63). Expression of ppIAPP led to higher accumulation of the dye as compared to the untreated cells, revealing a higher membrane potential, which was reduced by the treatment of cells with urolithin B (**Figure 6A**).

Mitochondrial damage is closely related to the accumulation of reactive oxygen species (ROS) (64). Hence, ROS levels were monitored by flow cytometry using dihydroethidium (DHE) fluorescence. ppIAPP expression was shown to increase ROS and urolithin B reduced it to control levels (**Figure 6B**). A genetic approach was also used to test this further, by using a mutant devoid of *YAP1* gene required for the transcriptional activation of oxidative stress response genes (65). Urolithin B-mediated protection was lost in the *yap1* mutant (**Figure 6C**) indicating that remediation of oxidative stress may also contribute to urolithin B bioactivity against IAPP proteotoxicity.

### Urolithin B changes the growth rate of cells

To elucidate how the modulation of IAPP aggregation and the described protective pathways by urolithin B impacts on cell growth, we first followed the growth of ppIAPP-expressing cells in the presence or absence of urolithin B for 24 h (**Figure 6D**). In line with the protective role of urolithin B towards IAPP aggregation and improved cellular response to a proteotoxic insult, the small molecule increased ppIAPP-expressing cell culture growth by ~ 17%. Although urolithin B seems to slightly decrease the growth rate of the cells, as concluded by the increase in doubling time, the final biomass of cultures and the area under the curve were significantly increased indicating an improved cellular function. As transcriptomics pointed cell cycle as one of the mechanisms affected by urolithin B, we evaluated the DNA content using PI staining to determine the distribution of cells in cell cycle phases. Quantitative data showed that urolithin B-treated cells had reduced distribution in the G0/G1 but significantly increased the percentage of S-G2/M phases (**Figure 6E**), suggesting that cells exposed to urolithin B remain actively growing and dividing for longer.

## Discussion

Diabetes is a multifactorial disease with complex molecular intervenients. IAPP is one of those key players and the consequences of its pathological aggregation disturb virtually all β-cell functions. Targeting this polypeptide hormone to hamper toxic oligomer formation and to avert the known effects on triggering and/or worsening diabetes is an appealing strategy. (Poly)phenols have been largely explored as a group of phytochemicals with potential to intervene in numerous diseases. Several molecules have been put forward as effective in suppressing IAPP aggregation (66), namely myricetin (67), resveratrol (24), and EGCG (20). Different (poly)phenols seem to act differently towards IAPP. On one hand, some molecules are described to interact directly with the protein and prevent its aggregation or even disassembled preformed fibrils (18). On the other hand, some molecules enhance cellular mechanisms, such as protein clearance, that help mitigating the intracellular effects of IAPP (14).

Our screening strategy was set-up to identify small molecules, among (poly)phenol metabolites, targeting IAPP pointed out urolithins as the most promising ones. The bioactivity properties of urolithins have been explored in multiple areas including neurodegenerative diseases, cancer, aging, among others (53, 68–73). From the three urolithins described (A, B and C), our study converged on urolithin A and B. Both small molecules were predicted to have as good, or higher, binding affinities than myricetin or EGCG that were previously described by molecular docking to be effective in vitro in interfering with IAPP aggregation and protecting cells from its toxicity (21, 48). Although this approach has limitations as it uses a rigid structure and the scoring is a simplified approach in the evaluation of the true free energy of binding, nevertheless herein it correctly guided the prioritization of the evaluation of molecules, and provided the correct results confirmed by the biological assays.

In the ppIAPP-expressing *S. cerevisiae* model, partial processing of ppIAPP by endogenous convertases leads to the accumulation of highly amyloidogenic immature IAPP forms thereby promoting toxic protein aggregation (27). In this way, the effectiveness of the molecules to inhibit aggregation and sustain cell viability could be investigated in a cellular milieu comprising a blend of immature and mature IAPP forms, mimicking what happens *in vivo* in the context of diabetes (10–12). In the yeast model, ppIAPP expression and aggregation cause marked cellular toxicity, thus providing a simple readout to assess molecules bioactivity by easily monitoring PI staining by flow cytometry. Unexpectedly, only urolithin B conferred cell protection against IAPP proteotoxicity, regardless of efforts in increasing urolithin A concentration and cell permeability (**Figure 1C** and **Supplementary Figure 1C**).

Encouraged by the results indicating that urolithin B performs efficiently in cells, we moved on the characterization of the anti-aggregating properties of the small molecule. Unequivocally, urolithin B interferes with the first stages of aggregation, promoting an extended lag phase (**Figure 2A**). It is visible that at 10 h urolithin B causes the protein to present in a more globular shape instead of the long fibrils seen in the untreated condition (**Figure 2B**). From **Figure 2C** it is also visible that until 24 h there are less species bigger than 2 µm (membrane pore size) (35) in the treatment condition. This indicates that oligomeric and pre-amyloid species formed are smaller than in the control condition and less structurally complex. Similar behaviors have been seen for other (poly)phenols, such as EGCG and resveratrol generating off-pathway IAPP species (21, 74). This is in agreement with the *in silico* data showing that urolithin B may interact directly with the IAPP and the oligomeric/pre-amyloid species formed possibly by stabilizing the hydrophobic pocket of the protein and, therefore, its structure.

A similar effect towards protein aggregation seems to happen in the cellular *milieu*. The number of yeast cells with proteinaceous aggregates were significantly reduced in the presence of urolithin B and the area of these intracellular aggregates seems to decrease (**Figure 3A**). The biochemical nature of these aggregates was also distinct as they were shown to be composed of less insoluble and more soluble protein (**Figure 3C**) and to have less SDS resistant high molecular weight species (**Figure 3B**). This may be due to the predicted interaction of urolithin B with aromatic sidechains of IAPP (**Figure 1B**), reducing self-association and aggregation.

Remarkably, the cell assays unveiled that urolithin B protection could be conveyed by more than the direct effect on IAPP structure. In the transcriptomic analysis, there were significantly altered genes in the control cells incubated with urolithin B. Furthermore, the heatmap of significantly different expressed genes shows that urolithin B shifts the expression pattern in respect to ppIAPP-induced changes (**Figure 4A**). Evaluation of the functional relevance of key proteostasis pathways indicated by genome wide analysis corroborates the importance of autophagy in urolithin B-mediated protection. Autophagy is a highly conserved mechanism that protects cells against proteotoxic insults. Reports of the accumulation of IAPP oligomers in β-cells indicates that these mechanisms are either defective or overwhelmed in the disease context (16, 75). Particularly, a study using human IAPP transgenic rat islets showed increased number of autophagosomes, accumulation of p62 and formation of p62-positive cytoplasmatic inclusions due to impaired lysosomal degradation (58). Urolithin B performs as efficiently as the autophagy inducer rapamycin in reducing the cell toxicity caused by IAPP (**Figure 5A**) and loses its protection capacity in an autophagy-deficient mutant (**Figure 5B**). This indicates that autophagy may play a key role in urolithin B protection against IAPP–induced toxicity. Autophagy enhancers were previously shown to protect the rodent cell line INS 832/13 from IAPP-induced apoptosis (58). Another study shows that, even with no aggregation, the cellular IAPP content is modulated by autophagy in a cell line and human islets (76). Indeed, the capacity of the cell to clear IAPP was improved in the presence of urolithin B (**Figure 5C**) further supporting the action of urolithin B in the improvement of IAPP clearance and thereby reducing the protein burden.

The mitochondria play a central role in the control of sustained phase of insulin secretion (77, 78) and β-cell mass. IAPP pathological effects were described to cause altered mitochondrial dynamics, with fragmented organelles and reduced mitophagy (79). Accordingly, one of enriched pathways in the transcriptomic analysis was oxidative phosphorylation, which is dependent on mitochondrial membrane potential. Furthermore, our data indicates that IAPP also caused an increase in mitochondrial membrane potential. As mitochondrial function and oxidative stress are closely related, we assessed ROS levels and found an increase in ppIAPP-expressing cells. These data are aligned with previous studies in INS-1 pancreatic β-cells showing increased levels of ROS and high mitochondrial membrane potential in IAPP-expressing cells compared with controls (80). Noteworthy, our data show for the first time that in IAPP-damaged cells, urolithin B significantly reduces both parameters in line with previous reports indicating that urolithin B modulates antioxidant responses in cardiomyocytes (81) and microglia (82).

IAPP expression causes faults in the cell physiology and metabolism and our data show that urolithin B protects cells by preventing the formation of toxic intracellular aggregates and by impacting on cellular mechanisms compromised by IAPP proteotoxicity. Thus, the expected effect of urolithin B-mediated protection is the amelioration of cell proliferation. In fact, the final biomass of ppIAPP-expressing cells treated with urolithin B was significantly increased. This is supported by the higher percentage of S-G2/M phases in urolithin B treated cells (**Figure 6E**), indicating an enhanced proliferating activity mediated by urolithin B treatment possibly as consequence of improved cellular function.

**Figure 7:**
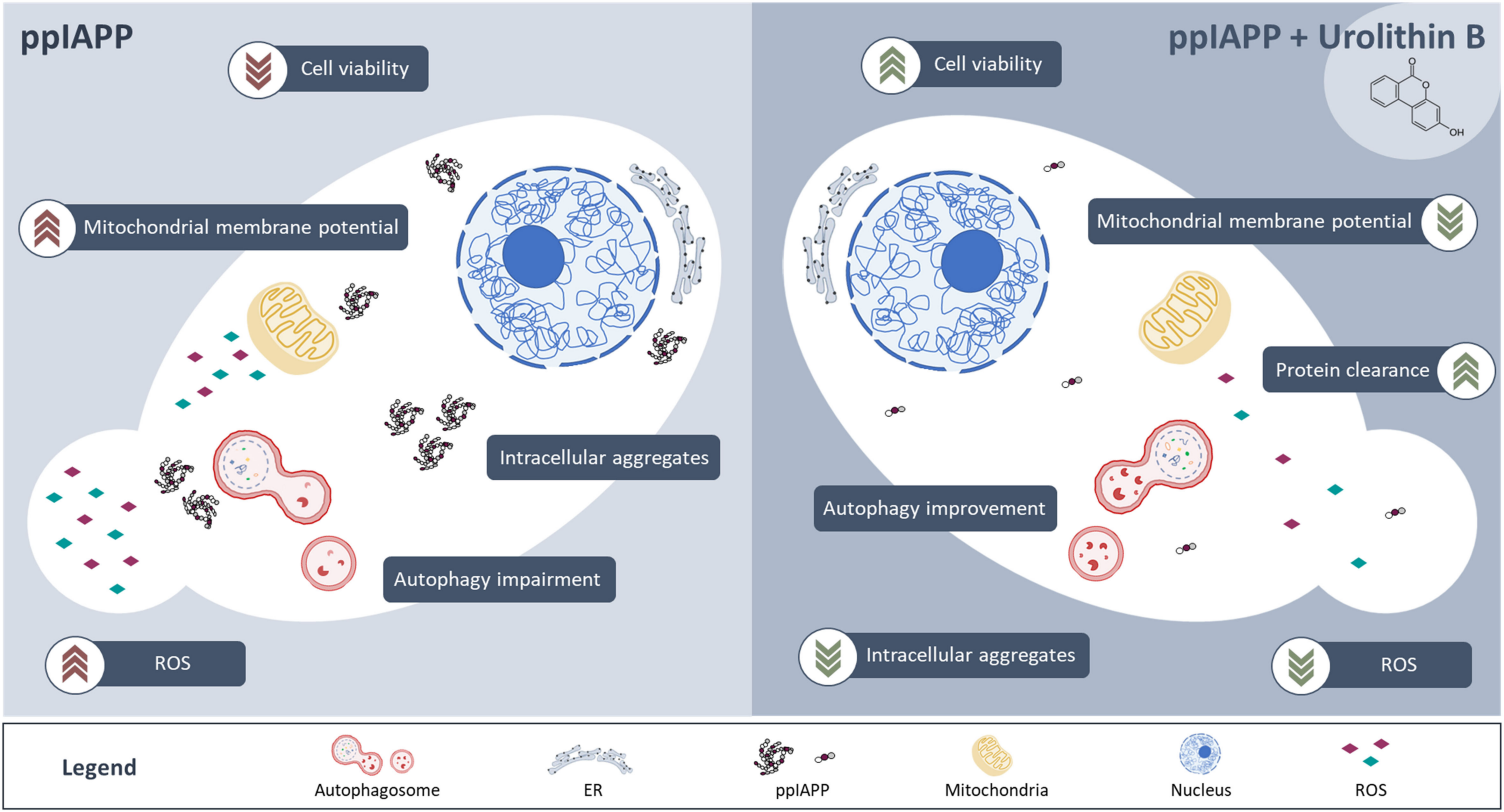
IAPP expression in yeast leads to the deposition of intracellular aggregates, which in turn interfere with multiple cellular functions such as autophagy, mitochondrial membrane potential homeostasis and redox homeostasis culminating with reduced cell viability and growth (left panel). Treatment of cells with urolithin B protects against IAPP proteotoxicity, preventing the accumulation of toxic intracellular aggregates, improving cellular functions and cell viability (right panel).

In summary, this study unveils urolithin B as a dual-effect small molecule targeting IAPP proteotoxicity, summarized in **Figure 7**. On one hand, by stabilizing the monomer structure it interferes in the early stages of IAPP aggregation. Urolithin B reshapes the low-order oligomeric species and consequently the pre-amyloid fibers leading to the formation of less toxic aggregates. Furthermore, it also improves the fitness of the cell, through different mechanisms including autophagy, maintenance of mitochondrial function, and redox homeostasis. Exploration of urolithin B as a promising small molecule to prevent IAPP proteotoxicity, with implications for diabetes, is still in its infancy. Further studies are still required to further characterize urolithin B bioactivity in superior models and to validate its therapeutic potential *in vivo*. As urolithin B results from the colonic metabolism of dietary ellagic acid in the human body, its bioactivity towards the mitigation of IAPP proteotoxicity has the potential to be explored from a nutritional, nutraceutical, and pharmacological perspectives.

## Data availability statement

The data presented in this study is deposited and publicly available at https://www.ncbi.nlm.nih.gov/geo/query/acc.cgi?acc=GSE218152.

## Author contributions

Conceptualization, RM; methodology, AR, SF, RM; investigation, AR, SF, VP, ML-d-S, JB, NS, DJVAS; data curation, RM and CS; writing—original draft preparation, AR; writing—review and editing, AR, SF, VP, ML-d-S, JB, NS, CS, RM, DJVAS. All authors have read and agreed to the published version of the manuscript.
