# Supplementary material for: Urolithin B: Two-way attack on IAPP proteotoxicity with implications for diabetes
Source: Front Endocrinol (Lausanne). 2022 Dec 15;13:1008418. doi: 10.3389/fendo.2022.1008418 (PMC9797523; doi:10.3389/fendo.2022.1008418)
Supplement: Supplementary file 1 [file DataSheet_1.docx]

Supplementary Material

# Supplementary Figures

A

**
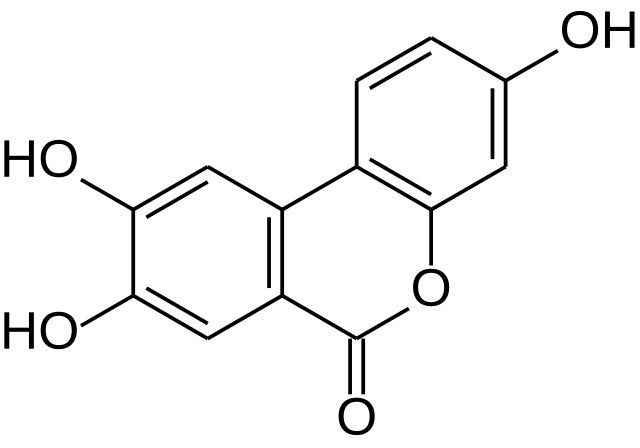

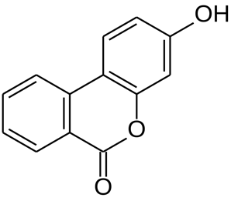
**

**
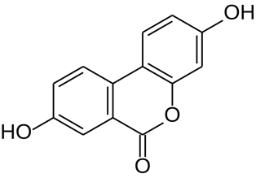
**

Urolithin A

Urolithin B

Urolithin C

**
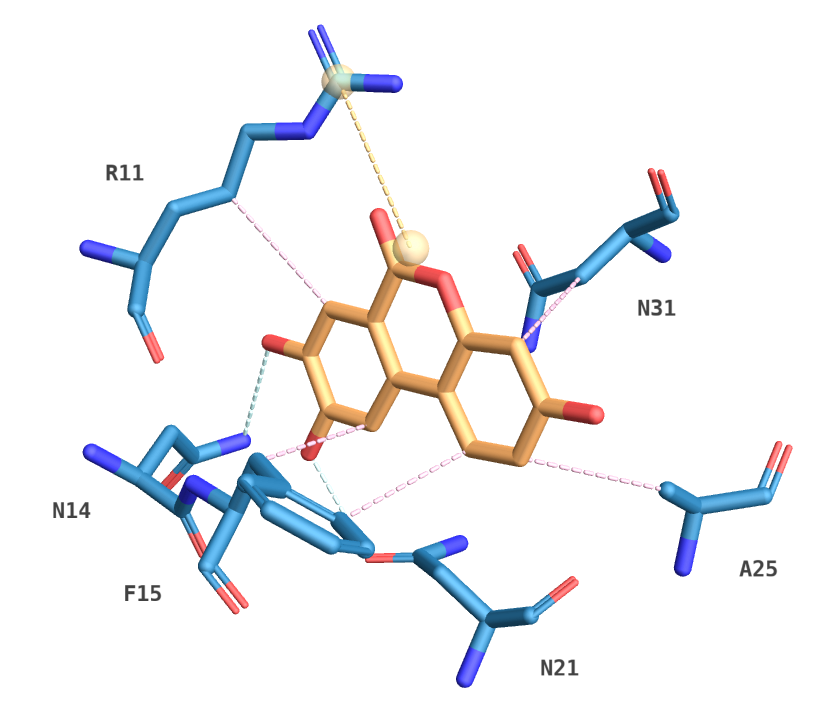

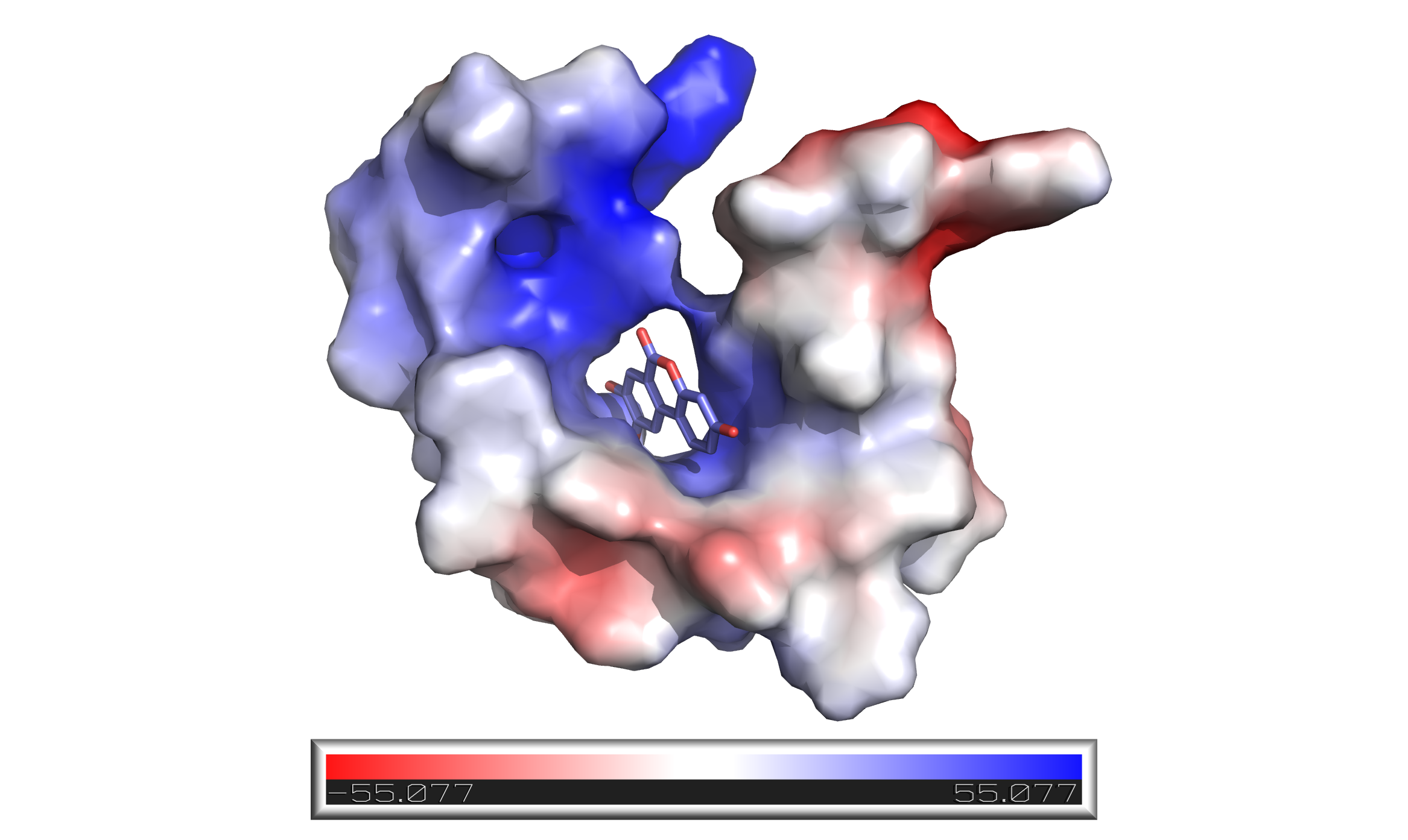
**

B

C

D

Supplementary Figure 1: (A) Chemical structures of urolithin A, B and C. (B) Vacuum electrostatics of urolithin C docking in IAPP using Pymol (left panel). Predicted bond analysis of urolithin C with IAPP amino acid residues (right panel). Green – π-stacking. Pink – Hydrophobic interactions. Blue – hydrogen bonds. Yellow – Salt bridges. (C) The frequency of propidium iodide (PI) positive cells assessed by flow cytometry in cells expressing ppIAPP and the respective control in the presence of urolithin A and SDS. (D) The frequency of PI positive cells in the presence of epigallocatechin gallate (EGCG). The values represent mean ± SD from at least three independent experiments. Statistical differences are denoted as ^**^*p* < 0.01 vs. the control condition. E – cells bearing the empty vector. I – cells expressing ppIAPP. SDS - Sodium dodecyl sulfate


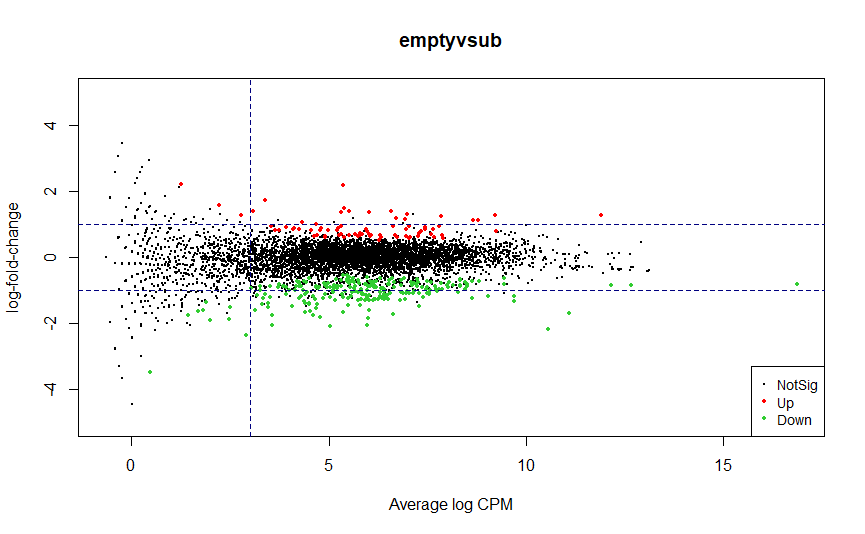
(A) (B)

E vs I

E vs EU

E vs IU


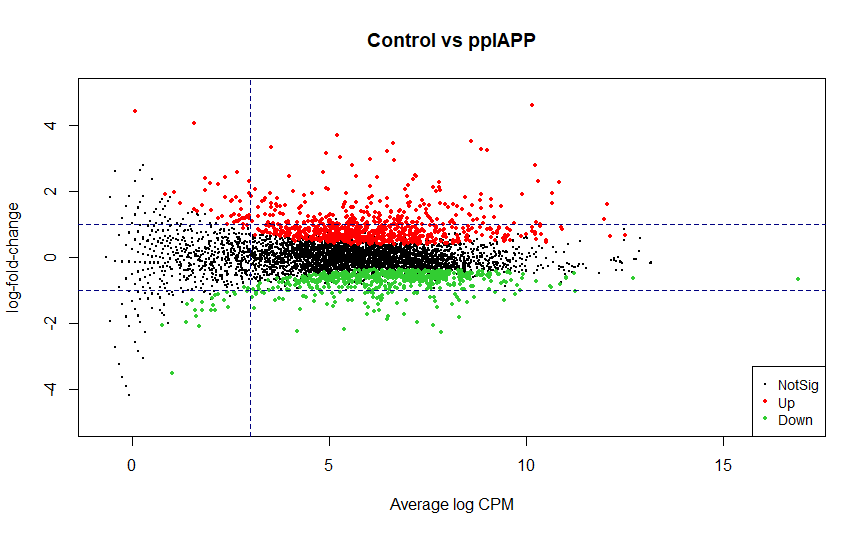


I vs IU


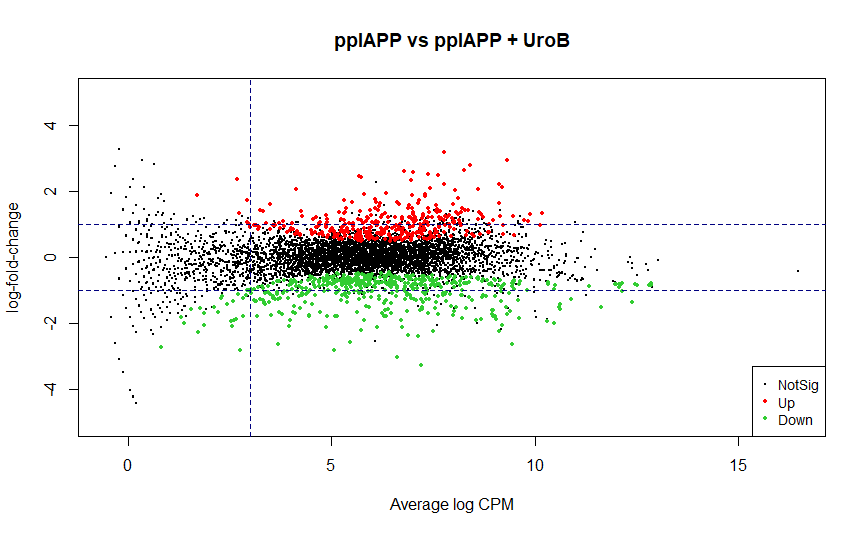


Supplementary Figure 2: (A) Funnel plots of differently expressed genes in the different comparisons. (B) Functional enrichment analysis obtained from GeneCodis3 of differently expressed genes between control and ppIAPP expressing cells with or without UroB. The list of genes up and down regulated of the explored pathways is also depicted. E – cells bearing the empty vector. I – cells expressing ppIAPP. IU – cells expressing ppIAPP incubated with UroB.

# Supplementary Tables

**Supplementary Table 1: Strains used in this study.**

| **Strain** | **Genetic background** |
| --- | --- |
| BY 4741 | *MATa his3Δ1 leu2Δ0 met15Δ0 ura3Δ0* |
| *atg8* | *MATa ura3Δ0 leu2Δ0 his3Δ1 met15Δ0 YBL078c::kanMX4* |
| *atg11* | *MATa ura3Δ0 leu2Δ0 his3Δ0 met15Δ0 YPR049c::kanMX4* |
| *atg32* | *MATa ura3Δ0 leu2Δ0 his3Δ0 met15Δ0 YIL146c::kanMX4* |
| *yap1* | *MATa ura3Δ0 leu2Δ0 his3Δ0 met15Δ0 YML007w::kanMX4* |

**Supplementary Table 2: Primers used in qRT-PCR for RNAseq validation of target genes.**

| **Gene** | **Forward** | **Reverse** |
| --- | --- | --- |
| *ATG5* | TCGGTCAACGAAGCTCGAAA | ATGAGCGGTATATGTCGCGG |
| *ATG17* | GCAGAGCAGTTTAGCAGGGA | ACAGCTCCTCACGTTCATCG |
| *QCR9* | GCAGGTGCCTTTGTTTTCCA | ATCATCGTCGTCTCCATCGC |
| *COX6* | GGGCTGCCAGAAGAGTCAAT | AGGGAACGCCCAATTCTTGT |
| *SIL1* | CCCCGGTGGTTTGGACATTA | TCACCAGGCGATGCTTTCAT |
| *ERO1* | AGGACTGGGATACACTGCCT | CGTCATCGCTATCATCCGCT |
| *PDA* | TGACGAACAAGTTGAATTAGC | TCTTAGGGTTGGAGTTTCTG |

**Supplementary Table 3: Vina scores (kcal mol^-1^) to all the tested ligands.**

| **Class** | **IUPAC Name** | **Other names** | **Vina score** |
| --- | --- | --- | --- |
| **Cinnamic and Phenylpropionic** | (2E)-3-(3,4-dihydroxyphenyl)prop-2-enoic acid | Caffeic Acid | -5.5 |
|  | 3-(4-hydroxyphenyl)propanoic acid | Phloretic Acid | -5.1 |
|  | 3-(2,4-dihydroxyphenyl)propanoic acid | DPPA acid | -4.9 |
|  | 3-(3-hydroxyphenyl)propanoic acid | - | -5.2 |
|  | 3-(3,4-dihydroxyphenyl)propanoic acid | Dihydrocaffeic acid | -6.1 |
|  | 3-(4-Hydroxy-3-methoxyphenyl)propionic acid | Hydroferulic acid | -5.2 |
| **Class** | **IUPAC Name** | **Other names** | **Vina score** |
| **Flavonols and catechins** | (2R,3R)-2-(3,4-dihydroxyphenyl)-3,4-dihydro-2H-chromene-3,5,7-triol | (-)-Epicatechin | -6.7 |
|  | (2R,3S)-2-(3,4-dihydroxyphenyl)-3,4-dihydro-2H-chromene-3,5,7-triol | (+)-Catechin | -6.9 |
|  | 3,5,7-trihydroxy-2-(4-hydroxy-3-methoxyphenyl)chromen-4-one | Isorhamnetol | -6.8 |
|  | 3,5,7-trihydroxy-2-(4-hydroxyphenyl)chromen-4-one | Rhamnolutin | -6.9 |
|  | 3,5,7-trihydroxy-2-(3,4,5-trihydroxyphenyl)chromen-4-one | Myricitin | -6.8 |
|  | 2-(3,4-dihydroxyphenyl)-3,5,7-trihydroxychromen-4-one | Quercitin | -6.8 |
| **Class** | **IUPAC Name** | **Other names** | **Vina score** |
| **Gallic acid and hydroxybenzoics** | 2,4-dihydroxybenzoic acid | Benzoic acid | -4.9 |
|  | 2-[(2-hydroxybenzoyl)amino]acetic acid | 2-Hydroxyhippuric acid | -5.6 |
|  | 3,4-dihydroxybenzoic acid | Protocatehuic acid | -4.9 |
|  | 3,4-dimethoxybenzoic acid | Veratric acid | -5.1 |
|  | 6,7,13,14-tetrahydroxy-2,9-dioxatetracyclo[6.6.2.04,16.011,15]hexadeca-1(15),4,6,8(16),11,13-hexaene-3,10-dione | Ellagic acid | -6.8 |
|  | 3,5-dihydroxybenzoic acid | alpha-Resorcylic acid | -4.8 |
|  | 3-hydroxybenzoic acid | m-Salicylic acid | -4.7 |
|  | 3-methoxybenzoic acid | m-Anisic acid | -4.8 |
|  | 2-[(4-hydroxy-3-methoxybenzoyl)amino]acetic acid | Vanilloylglycine | -5.3 |
|  | 3,4,5-trimethoxybenzoic acid | - | -5.0 |
|  | 4-hydroxybenzoic acid | - | -4.6 |
|  | 2-[(4-hydroxybenzoyl)amino]acetic acid | P-hydroxyhippuric acid | -5.3 |
|  | 4-methoxybenzoic acid | p-Anisic acid | -4.7 |
|  | benzoic acid | Dracylic acid | -4.5 |
|  | 3,4,5-trihydroxybenzoic acid | Gallic acid | -5.0 |
|  | 2-benzamidoacetic acid | Hippuric acid | -5.5 |
|  | 2-(4-hydroxy-3-methoxyphenyl)acetic acid | Vanillacetic acid | -5.1 |
|  | 3-hydroxy-4-methoxybenzoic acid | Isovanillic acid | -4.9 |
|  | 4-hydroxy-3,5-dimethoxybenzoic acid | Syringic acid | -5.0 |
|  | 4-hydroxy-3-methoxybenzoic acid | Vanillic acid | -4.9 |
| **Class** | **IUPAC Name** | **Other names** | **Vina score** |
| **Hydroxybenzoics and phenylacetic acids** | 2-(2-hydroxyphenyl)acetic acid | - | -4.7 |
|  | 3,4-dihydroxybenzaldehyde | - | -4.8 |
|  | 2-(3,4-dihydroxyphenyl)-2-hydroxyacetic acid | - | -5.2 |
|  | 2-(3,4-dihydroxyphenyl)acetic acid |  | -5.4 |
|  | 2-(3,4-dimethoxyphenyl)acetic acid | Homoveratric acid | -4.9 |
|  | 2-hydroxy-2-(3-hydroxyphenyl)acetic acid | - | -5.1 |
|  | 2-(3-hydroxyphenyl)acetic acid | - | -5.1 |
|  | (2S)-2-hydroxy-2-(4-hydroxy-3-methoxyphenyl)acetic acid | Vanillomandelic acid | -5.2 |
|  | 2-(4-hydroxyphenyl)acetic acid | - | -4.9 |
|  | 2-[[2-(4-hydroxyphenyl)acetyl]amino]acetic acid | - | -5.7 |
|  | 2-[(4-methoxyphenyl)methylamino]acetic acid | - | -5.0 |
|  | 2-(4-methoxyphenyl)acetic acid | Homoanisic acid | -4.9 |
|  | 3,5-dihydroxy-4-methoxybenzoic acid | - | -5 |
| **Class** | **IUPAC Name** | **Other names** | **Vina score** |
| **Phenylacetic acids** | 2-hydroxy-2-[4-hydroxy-3-(trideuteriomethoxy)phenyl]acetic acid | - | -5.2 |
|  | 2-hydroxy-2-phenylacetic acid | Phenylglycolic acid | -4.9 |
| **Class** | **IUPAC Name** | **Other names** | **Vina score** |
| **Simple phenols** | 4-methylbenzene-1,2-diol | Homocatechol | -4.6 |
|  | benzene-1,2-diol | Pyrocatechin | -4.1 |
|  | 2,4-dihydroxybenzaldehyde | - | -4.7 |
|  | 2,4,6-trihydroxybenzaldehyde | Phloroglucinol aldehyde | -4.4 |
|  | benzene-1,3,5-triol | Phloroglucinol | -4.1 |
|  | benzene-1,2,3-triol | Pyrogallol | -4.2 |
|  | benzene-1,3-diol | Resorcinol | -4.1 |
| **Class** | **IUPAC Name** | **Other names** | **Vina score** |
| **Urolithins** | 3,8,9-trihydroxybenzo[c]chromen-6-one | Urolithin C | -7.5 |
|  | 3,8-dihydroxybenzo[c]chromen-6-one | Urolithin A | -7.0 |
|  | 3-hydroxybenzo[c]chromen-6-one | Urolithin B | -7.0 |

**Supplementary Table 4: Results of the Vinardo and MOE scoring functions.**

| **Molecule** | **Score (kcal mol^-1^)** | |
| --- | --- | --- |
|  | **Vinardo** | **MOE** |
| Urolithin A | -5.8 | -4.8 |
| Urolithin B | -5.9 | -4.5 |
| EGCG | -5.8 | -6.2 |
| Resveratrol | -5.7 | -5.0 |
| Myricetin | -5.7 | -5.4 |
| Aspirin | -4.5 | -4.6 |
| Inositol | -3.6 | -4.2 |

**Supplementary Table 5: Positive and negative controls used in the docking simulations.**

| **PubChem ID** | **IUPAC Name** | **Other names** | **Vina score** |
| --- | --- | --- | --- |
| **Positive controls** | | | |
| 5281672 | 3,5,7-trihydroxy-2-(3,4,5-trihydroxyphenyl)chromen-4-one | Myricetin | -6.8 |
| 445154 | 5-[(E)-2-(4-hydroxyphenyl)ethenyl]benzene-1,3-diol | Resveratrol | -6.4 |
| 65064 | (2R,3R)-5,7-dihydroxy-2-(3,4,5-trihydroxyphenyl)-3,4-dihydro-2H-1-benzopyran-3-yl 3,4,5-trihydroxybenzoate | (-)-epigallocatechin gallate | -7.1 |
| **Negative controls** | | | |
| 892 | (1R,2R,3S,4S,5R,6S)-Cyclohexane-1,2,3,4,5,6-hexol | Myo-Inositol | -4.5 |
| 2244 | 2-acetyloxybenzoic acid | Aspirin, Acetylsalicylic Acid | -5.1 |

**Supplementary Table 6: SwissADME characteristics of urolithin A and urolithin B regarding the putative likelihood of using the compounds as drugs**

|  | **Urolithin B** | **Urolithin A** |
| --- | --- | --- |
| **Water solubility** | | |
| ESOL Class | Soluble | Soluble |
| Ali Class | Soluble | Soluble |
| Silicos-IT class | Moderately soluble | Moderately soluble |
| **Pharmacokinetics** | | |
| GI absorption | High | High |
| BBB permeant | Yes | Yes |
| Pgp substrate | No | No |
| CYP1A2 inhibitor | Yes | Yes |
| CYP2C19 inhibitor | No | No |
| CYP2C9 inhibitor | No | No |
| CYP2D6 inhibitor | No | No |
| CYP3A4 inhibitor | No | No |
| **Druglikeness** | | |
| Lipinski #violations | 0 | 0 |
| Ghose #violations | 0 | 0 |
| Veber #violations | 0 | 0 |
| Egan #violations | 0 | 0 |
| Muegge #violations | 0 | 0 |
| Bioavailability Score | 0.55 | 0.55 |
